# Physical Fitness and the Level of Pain Intensity in Adolescents: A School-based Study

**DOI:** 10.3390/ijerph16132410

**Published:** 2019-07-06

**Authors:** Martin Zvonar, Mario Kasović, Lovro Štefan

**Affiliations:** 1 Faculty of Sport Studies, Masaryk University, 62 500 Brno, Czech Republic; 2 Department of General and Applied Kinesiology, Faculty of Kinesiology, University of Zagreb, 10 000 Zagreb, Croatia

**Keywords:** secondary-school students, fitness, pain, associations

## Abstract

*Background*: The main aim of the study was to explore the association between objectively measured physical fitness and the level of pain intensity. *Methods*: In this cross-sectional study, we included 1036 adolescents (m_age_ ± SD = 16.3 ± 1.1 years; m_height_ ± SD = 1.74 ± 0.1 m; m_weight_ ± SD = 64.7 ± 12.4 kg; m_body-mass index_ ± SD = 21.3 ± 3.0 kg/m^2^) from 11 secondary schools located in the city of Zagreb (Croatia). Physical fitness was determined by using waist circumference, sit-ups in 1 min, standing long jump and sit-and-reach tests. Overall physical fitness index was calculated by summing the *z*-score values of each physical fitness test. The level of pain intensity was assessed with the Numeric Pain Rating Scale, a one-dimensional measure of pain intensity. Associations were calculated with correlation analyses. *Results*: In boys, pain intensity was associated with sit-ups in 1 min (*r* = −0.16, *p* < 0.001), standing long jump (*r* = −0.14, *p* = 0.003) and overall physical fitness index (*r* = −0.13, *p* = 0.004), while no significant associations with waist circumference (*r* = 0.04, *p* = 0.438) and sit-and-reach test (*r* = −0.01, *p* = 0.822) were observed. In girls, pain intensity was associated with standing long jump (*r* = −0.17, *p* < 0.001) and overall physical fitness index (*r* = −0.10, *p* = 0.018), while no significant associations with waist circumference (*r* = 0.01, *p* = 0.735), sit-ups in 1 min (*r* = −0.06, *p* = 0.126) and sit-and-reach test (*r* = −0.05, *p* = 0.232) were observed. When we adjusted for self-rated health, sleep duration, smoking status, alcohol consumption, screen-time and psychological distress, similar associations remained. *Conclusions*: Our study shows a weak association between physical fitness and pain intensity in a large sample of adolescents. Although a cross-sectional design, health-professionals should use physical fitness as a screening tool to assess the level of pain intensity.

## 1. Introduction

Pain complaints have become one of the leading causes for people seeking medical attention worldwide [1]. It has been estimated that 1/5 of adults suffer from pain [2]. Studies have shown that higher pain levels have a long-term negative impact on overall health [3].

In recent years, more attention has been given in exploring the prevalence of pain in children and adolescents [4]. Annual prevalence of headache, stomachache and backache is 54.1%, 49.8% and 37%, respectively, and these types of pain frequently co-exist [4]. It is worthwhile to note that pain experienced in childhood and adolescence is a significant predictor of pain in adulthood [5].

For many years, a main prescription for pain relief was rest and physical inactivity [6]. Although numerous non-modifiable factors may contribute to an increased risk of pain (female gender, older age, lower socioeconomic status), physical activity and exercise should be prescribed having specific benefits in reducing the severity of pain [6]. However, a recent longitudinal study conducted among Danish adolescents has shown that 10% of participants with the highest proportion of the day spent in objectively measured vigorous physical activity are at increased risk of reporting spinal pain at 2 year follow-up period [7]. Another study aiming to determine if adolescents across weight status report pain relief following high-intensity aerobic exercise showed no significant association between physical activity/exercise nor physical fitness and reported pain [8]. Nevertheless, higher level of physical activity can be a significant contributor of reporting less pain [9].

According to the aforementioned, the majority of the studies have explored the association between physical activity/exercise and pain [7,8,9]. A major part of physical activity is physical fitness [10]. It is defined as ‘a measure of the capacity to perform physical activity and/or physical exercise that integrates of the majority of the bodily functions (skeleton-muscular, cardio-respiratory, hemato-circulatory, endocrine-metabolic and psycho-neurological) involved in body movements [10]. Although significant, studies have shown only weak to moderate association between physical activity and physical fitness pointing out that those two factors may contribute differently on the level of pain. As for the pain, the level of physical fitness in childhood/adolescence often persists later in adulthood [11]. Studies examining the association between physical fitness and general level of pain in adolescents are scare. Since both factors (low fitness level/high level of pain) contribute on overall health [12,13], it is necessary to explore whether physical fitness is a significant predictor of pain. Therefore, the main aim of the study was to explore the association between objectively measured physical fitness and the level of pain in a sample of 15–18 year old adolescents.

## 2. Materials and Methods

### 2.1. Study Participants

In this cross-sectional study, participants were secondary-school students. At the first stage, we randomly selected 11 (eight grammar and three vocational) out of 86 secondary-schools currently operating in the city of Zagreb. At the second stage, we randomly selected one class representing each grade within the school (from 1st to 4th). Each class had ≈25 students. All students were considered healthy and were not affected by diseases. The selection criteria were: (1) active participation to physical education (PE) classes, (2) absence of injuries. According to the Croatian Bureau of Statistics for the year 2017 [14], there were 36 350 secondary-school students in total. Our sample size was estimated to be 1030 by using 95% confidence level and 3% margin error. At the beginning, we recruited 1247 participants. Of these, 136 did not provide full data and 75 were absent when the study was conducted. Our final sample was based on 1036 secondary-school students (m_age_ ± SD = 16.3 ± 1.1 years, meadian_age_ [interquartile range 25th–75th] = 16.0 [15.0–17.0] years; m_height_ ± SD = 1.74 ± 0.1 m; median_height_ [interquartile range 25th–75th] = 1.7 [1.6–1.8] m; m_weight_ ± SD = 64.7 ± 12.4 kg; median_weight_ [interquartile range 25th–75th] =63.0 [55–71] kg; m_body-mass index_ ± SD = 21.3 ± 3.0 kg/m^2^, median_body-mass index_ [interquartile range 25th–75th] = 20.9 [19.4–22.6] kg/m^2^ 55.3% girls). After the selection of each school and class, we contacted physical education teachers to help us organize the study and obtain the approval of the principal. The measurement protocol for the study lasted from January to March 2019. For ≈25 students, it took us 30 min in each physical education class to collect the data. Before the study began, all students had got familiar with aims, hypotheses and benefits for participation in the study. There were no risks for participants with valid data and included in further analyses. All procedures performed in this study were in accordance with the Declaration of Helsinki and approved by the Institutional Review Board of the Faculty of Kinesiology (Ethics code: 02/2019). Also, all participants and their parents/guardians provided written informed consent for participation in the study.

### 2.2. The Numeric Pain Rating Scale

The Numeric Pain Rating Scale was used to assess pain intensity in the last 24 h [15]. It is scored from 0 (no pain) to 10 (worst possible pain). Previous studies have shown acceptable reliability and validity of the scale when used with children and adolescents [15].

### 2.3. Physical Fitness

We used EUROFIT Battery Fitness Test to assess the level of physical fitness in adolescents. These tests are considered reliable and valid instruments to measure the level of physical fitness in children and adolescents [16]. Waist circumference, standing long jump, sit-ups in 1 min and sit-and-reach test were chosen because of their mutual independence to the other [17]. Data were collected by two trained researches in order to guarantee the standard measurement methodology [17]. A brief explanation of each test is presented below:

### 2.4. Waist Circumference

Each participant stood still in a standing position. We used anthropometric tape placed horizontally midway between the lower rib margin and the iliac crest at the end of normal expiration [18].

### 2.5. Standing Long Jump

Each subject performed distance jumps from a standing start. While performing the jumps, the subjects were asked to bend their knees with their arms in front of them, parallel to the ground, then to swing both arms, push off vigorously and jump forward as far as possible, trying to land with their feet together and stay upright. The best out of two attempts was taken as the final score (expressed in centimeters) [19].

### 2.6. Sit-ups in 1 min

Trunk strength was assessed as the maximum number of sit-ups achieved in one minute. Children were seated on the floor, backs straight, hands clasped behind their neck, knees bent at 90° with heels and feet flat on the mat. They then lay down on their backs, shoulders touching the mat, and returned to the sitting position with their elbows out in front to touch their knees, keeping the hands clasped behind their neck the whole time. The total amount of correctly performed sit-ups in 60 s was the score [19].

### 2.7. Sit-and Reach Test

Sitting on the floor or a mat, legs straight under the angle of 90°, the person being tested reached forward with the arms (hands overlapping). The distance of reach was measured in centimeters using a measuring non-elastic tape attached on the floor [20].

### 2.8. Height, Weight and Body-Mass Index

Height and weight were collected using a portable stadiometer (stadiometer and balance beam, Seca, Hambur, Germany) with accuracy nearest to 0.5 cm and 0.2 kg in accordance to the International Society for the Advancement of Kinanthropometry [21].

### 2.9. Covariates

Self-rated health was assessed using a one-item question: ‘How would you rate your health?’ Answers were arranged as follows: (1) very poor, (2) poor, (3) fair, (4) good and (5) excellent. To assess sleep duration, we asked the participants to provide information on their hours of sleep during an average night [22]. Smoking status and alcohol consumption were based on their current cigarette smoking or consuming alcohol drinks (‘Yes’ and ‘No’ option). We asked the participants to provide the daily time they spent in watching television and using mobile-phone for fun. Other question coved the time spent in playing computer or mobile-phone games. We summed both times to get total screen-time [23]. Psychological distress was assessed using Kessler’s six-item questionnaire: (1) ‘How often during the past 30 days did you feel nervous?’, (2) ‘How often during the past 30 days did you feel hopeless?’, (3) ‘How often during the past 30 days did you feel restless or fidgety?’, (4) ‘How often during the past 30 days did you feel so depressed that nothing could cheer you up?’, (5) ‘How often during the past 30 days did you feel that everything was an effort?’ and (6) ‘How often during the past 30 days did you feel worthless?’ [24]. Each question is scored from 0 (none of the time) to 4 (all the time). The scores of each question are summed up between 0 and 24, with a lower score indicating a lower level of psychological distress.

### 2.10. Data Analysis

Basic descriptive statistics of the study participants are presented as mean and standard deviation (SD). Differences in waist circumference, sit-ups in 1 min, standing long jump and sit-and-reach test and within each pain intensity score were calculated by using univariate analysis of variance (ANOVA). To obtain overall physical fitness index, we calculated *z*-scores for each physical fitness test. Then, we summed *z*-score values. Correlation analysis (*r*) was used to determine the relationship between pain intensity and physical fitness. We additionally adjusted for self-rated health, sleep duration, smoking status, alcohol consumption, screen-time and psychological distress. Significance was set up at α ≤ 0.05, and it was two sided (2-sided). All the analysis were performed in Statistical Package for Social Sciences Software, ver. 22 (IBM Corp., Armonk, NY, USA).

## 3. Results

Basic descriptive statistics of the study participants are presented in Table 1. In boys, mean value of waist circumference was the highest in those who reported the highest pain intensity, although ANOVA relieved no significant differences between groups (*F* = 1.24, *p* = 0.262). However, mean values of both sit-ups in 1 min (*F* = 4.89, *p* < 0.001) and standing long jump (*F* = 2.52, *p* = 0.006) significantly decreased as the pain intensity score increased. No significant differences in sit-and-reach test were observed (*F* = 1.13, *p* = 0.341). In girls, ANOVA showed that the mean value of standing long jump significantly decreased as the pain intensity score increased (*F* = 3.00, *p* < 0.001), yet no significant differences in other physical fitness tests were observed (*p* = 0.298–0.813).

Correlations of all the study variables are presented in Table 2. In boys, the strongest inverse correlation was observed between the pain intensity and sit-ups in 1 min, followed by standing long jump and overall physical fitness index. Interestingly, no significant correlations between the pain intensity with waist circumference and sit-and-reach test were observed. Similar patterns were obtained after adjusting for self-rated health, sleep duration, smoking status, alcohol consumption, screen-time and psychological distress. In girls, the strongest inverse correlation was observed between the pain intensity and standing long jump, followed by overall physical fitness index. Other physical fitness tests were not significantly associated with pain intensity (*p* = 0.126–735). Similarly to boys, almost no changes in correlation coefficients were obtained after adjusting for self-rated health, sleep duration, smoking status, alcohol consumption, screen-time and psychological distress.

## 4. Discussion

The main aim of the study was to explore the association between objectively measured physical fitness and the level of pain in a sample of 15–18 year old adolescents. To the best of our knowledge, this is the first study examining the aforementioned associations in a sample of adolescents. However, previous study by Andersen et al. [25] conducted among 9413 secondary-school adolescents from Denmark showed that back pain was only significantly associated with low isometric muscle endurance, yet no associations to aerobic fitness, functional strength, flexibility or physical activity level were found. Another systematic review based on 17 studies showed that of high-quality studies with low methodological issues, there was strong evidence of no association between physical activity and neck pain in school-aged children [26]. The same study also presented conflicting results for the association between physical activity and low back pain in the same population [26]. Inconsistent to our findings, Stolzman et al. [8] also found no association between clinical pain and maximal aerobic exercise in adolescents with different weight status. Specifically, normal weight and overweight/obese adolescents experienced similar levels of exercise-induced hypoalgesia after maximal aerobic exercise, pointing out that nutritional status might be of less importance than physical fitness in determining quality of life in adolescents. In longitudinal design, another study presented results going to other direction, where 10% of adolescents with the highest proportion of the day spent in objectively measured vigorous physical activity were 44% more likely to report spinal pain at 2 years follow-up period [7]. Similar results were obtained in another longitudinal study of pain-free children that found that children who reported participating in sporting activities for more than 6 h a week were at increased risk for developing back pain one year later [27].

According to aforementioned, findings supporting the association between physical fitness and the level of pain are inconsistent. To date, systematic reviews have concluded that the literature with respect to the association between physical activity/fitness and pain is too heterogeneous with more research yet to be done in this field [6,26]. In our study, by using EUROFIT physical fitness tests and Numeric Pain Rating Scale, we were able to find significant, although very weak associations between a few aspects of physical fitness and pain intensity. However, by including the region and the type of the pain, the associations might have gone differently.

Our study has several strengths. First, we used a large sample of adolescents with low drop-out rate (17%) taken from randomly selected schools and classes, minimizing the risk of potential bias. Second, only two trained researchers conducted the measurement protocol, also minimizing the risk of potential measurement error. Third, we objectively measured the level of physical fitness by combining four tests and summing their *z*-score values. Fourth, we selected The Numeric Pain Rating Scale, which has been shown to have acceptable reliability and validity properties [15]. Finally, we adjusted for numerous socio-demographic and lifestyle covariates.

### Limitation of This Study

However, this study has some limitations. Due to a cross-sectional design, we cannot draw a conclusion regarding the inverse association that is higher level of pain led to lower physical fitness level and *vice versa*. Second, the level of pain is a subjective measure and the sensitivity to pain depends on individual differences in physiological, emotional and cognitive states [28]. Third, we did not include aerobic fitness (often estimated with a 20-m shuttle run test) as a part of overall physical fitness and it is possible that our results would have been different.

## 5. Conclusions

Our study shows a significant association between physical fitness and the level of pain in a sample of adolescents after adjusting for numerous covariates. Although we used a cross-sectional design, findings of our study might be a new addition to already existing and conflicting evidence examining the aforementioned associations. Although we presented weak associations between a few aspects of physical fitness and pain intensity, our results might be useful to health-professionals in screening for both physical fitness (fit vs. unfit individuals) and pain in a sample of adolescents.

## Figures and Tables

**Table 1:** Basic descriptive statistics of the study participants, Croatia (2019).

Study Variables	Pain Intensity (0–10)										
	0	1	2	3	4	5	6	7	8	9	10
**Boys (*N* = 463)**	**Mean (SD)**	**Mean (SD)**	**Mean (SD)**	**Mean (SD)**	**Mean (SD)**	**Mean (SD)**	**Mean (SD)**	**Mean (SD)**	**Mean (SD)**	**Mean (SD)**	**Mean (SD)**
Waist circumference (cm)	78 (9)	73 (3)	77(8)	78(9)	79 (11)	78 (15)	78 (9)	75(8)	81 (5)	75(2)	93(21)
Sit-ups in 1 min (#)	58(10)	79 (19)	58 (11)	53 (12)	56 (12)	57 (12)	54 (13)	52 (10)	51 (8)	49(9)	48(9)
Standing long jump (cm)	214(27)	230 (16)	213 (24)	212 (29)	205 (27)	203 (32)	206 (32)	201 (32)	206 (30)	217 (48)	149 (58)
Sit-and-reach test (cm)	62 (13)	64 (12)	60 (12)	64 (12)	61 (10)	62 (12)	64 (13)	59 (11)	59 (7)	65 (8)	49(30)
**Girls (*N* = 573)**	**Mean (SD)**	**Mean (SD)**	**Mean (SD)**	**Mean (SD)**	**Mean (SD)**	**Mean (SD)**	**Mean (SD)**	**Mean (SD)**	**Mean (SD)**	**Mean (SD)**	**Mean (SD)**
Waist circumference (cm)	69(7)	71 (11)	69(8)	70(6)	70(8)	69 (7)	69(6)	68(5)	70 (12)	72(7)	75(7)
Sit-ups in 1 min (#)	48 (19)	58 (13)	47 (11)	49 (8)	47(8)	47(8)	46 (10)	47 (11)	47 (12)	46(8)	44 (10)
Standing long jump (cm)	172 (26)	199 (21)	168 (18)	167 (18)	167 (22)	167 (17)	169 (18)	161 (19)	164 (26)	158 (28)	159 (18)
Sit-and-reach test (cm)	72 (15)	84 (10)	71 (13)	71 (14)	72 (12)	71 (12)	71 (10)	70 (16)	69 (12)	72 (15)	68 (15)

^#^ Number of repetitions.

**Table 2:** Correlation analysis between pain intensity and physical fitness in boys and girls, Croatia (2019).

Study Variables	Boys (*N* = 463)		Girls (*N* = 573)	
	Unadjusted *r* Coefficient (*p*-Value)	Adjusted *r* Coefficient (*p*-Value) *	Unadjusted *r* Coefficient (*p*-Value)	Adjusted *r* Coefficient (*p*-Value) *
Waist circumference (cm)	0.04 (0.438)	−0.003 (0.943)	0.01 (0.735)	0.01 (0.714)
Sit-ups in 1 min (#)	−0.16 (<0.001)	−0.15 (0.002)	−0.06 (0.126)	−0.01 (0.736)
Standing long jump (cm)	−0.14 (0.003)	−0.14 (0.003)	−0.17 (<0.001)	−0.10 (0.012)
Sit-and-reach test (cm)	−0.01 (0.822)	0.00 (0.993)	−0.05 (0.232)	−0.06 (0.184)
Overall physical fitness index (*z*-score)	−0.13 (0.004)	−0.14 (0.002)	−0.10 (0.018)	−0.08 (0.049)

* adjusted for self-rated health, sleep duration, smoking status, alcohol consumption, screen-time and psychological distress. *p* < 0.05
